# Frontal White Matter Alterations in Short-Term Medicated Panic Disorder Patients without Comorbid Conditions: A Diffusion Tensor Imaging Study

**DOI:** 10.1371/journal.pone.0095279

**Published:** 2014-04-30

**Authors:** Borah Kim, Jeong Hoon Kim, Min-Kyoung Kim, Kang Soo Lee, Youngki Kim, Tai Kiu Choi, Yun Tai Kim, Sang-Hyuk Lee

**Affiliations:** 1 Department of Psychiatry, CHA Bundang Medical Center, CHA University, Bundang-gu, Seongnam-si, Gyeonggi-do, Republic of Korea; 2 Department of Psychiatry, Bundang Jaesaeng Hospital, Bundang-gu, Seongnam-si, Gyeonggi-do, Republic of Korea; 3 Department of Psychiatry, CHA Gangnam Medical Center, CHA University, Gangnam-gu, Seoul, Republic of Korea; 4 Sujiyonsei Psychiatric Clinic, Suji-gu, Yongin-si, Gyeonggi-do, Republic of Korea; 5 Department of Applied Bioscience, College of Life Science, CHA University, Pocheon-si, Gyeonggi-do, Republic of Korea; University of Maryland, College Park, United States of America

## Abstract

The frontal cortex might play an important role in the fear network, and white matter (WM) integrity could be related to the pathophysiology of panic disorder (PD). A few studies have investigated alterations of WM integrity in PD. The aim of this study was to determine frontal WM integrity differences between patients with PD without comorbid conditions and healthy control (HC) subjects by using diffusion tensor imaging. Thirty-six patients with PD who had used medication within 1 week and 27 age- and sex-matched HC subjects participated in this study. Structural brain magnetic resonance imaging was performed on all participants. Panic Disorder Severity Scale and Beck Anxiety Inventory (BAI) scores were assessed. Tract-based spatial statistics (TBSS) was used for image analysis. TBSS analysis showed decreased fractional anisotropy (FA) in frontal WM and WM around the frontal lobe, including the corpus callosum of both hemispheres, in patients with PD compared to HC subjects. Moreover, voxel-wise correlation analysis revealed that the BAI scores for patients with PD were positively correlated with their FA values for regions showing group differences in the FA of frontal WM of both hemispheres. Altered integrity in frontal WM of patients with PD without comorbid conditions might represent the structural pathophysiology in these patients, and these changes could be related to clinical symptoms of PD.

## Introduction

Panic disorder (PD) affects approximately 4% of the general population [1] and is associated with significant social and vocational impairments [2]. Patients with PD have a considerably decreased quality of life over a long period of time [3]. However, a definite neurobiological etiology of PD has yet to be identified. Findings of various brain imaging studies point towards the existence of functional and structural neuroanatomical alterations in PD. Neuroimaging studies aimed at unraveling the circuits that mediate PD symptoms might help to understand and manage PD.

The fear network model of PD suggested by Gorman et al. [4] involves the amygdala, hippocampus, and frontal cortex. Several brain imaging studies have suggested the involvement of a cortical and subcortical network encompassing the amygdala, anterior cingulate cortex, and frontal cortex in human fear conditioning and extinction [5], [6]. This network has substantial overlap with the fear circuitry structures reported to exhibit aberrant activation patterns in a variety of anxiety disorders [7], [8] including PD [4], [9]. Further, it has been proposed that in anxiety disorders, the prefrontal areas cannot inhibit the hyperactivity of the anxiety-related neural circuit [10], which, in turn, could play a pathophysiological role in PD.

Some parts of the frontal lobe are implicated to be a part of the fear network and show altered functioning in PD. Previous functional magnetic resonance imaging (MRI) studies have reported the involvement of different regions of the frontal lobes in patients with PD. Being part of the panic circuitry [11], [12], [13], the prefrontal cortex modulates anxiety and other emotional behaviors. Hypofrontality in patients with a spontaneous panic attack was described in an iomazenil single-photon emission computerized tomography study [14]. Altered gamma-aminobutyric acid receptor binding, mainly in the frontal regions [15], might reflect abnormal neurotransmission in the frontal lobe of patients with PD. Prefrontal regions share extensive reciprocal connections with the amygdala, which indicates that prefrontal functioning can control the amygdala and inhibit anxiety.

Besides these functional alterations, structural abnormalities of the frontal lobe have also been demonstrated in PD. Gray matter (GM) volume reductions were observed in frontal structures such as the medial prefrontal, inferior frontal, and orbitofrontal cortices [16], [17], [18]. Previous studies have proposed that the frontal regions involve a top-down mechanism for regulating sensory stimuli from the temporal lobe to control anxiety or panic symptoms. Thus, both functional and structural alterations in the frontal cortex have been reported as neural correlates of PD.

Until now, a few imaging studies of WM have been conducted in patients with PD. Although the neural pathophysiology in psychiatric disorders is hard to assess, diffusion tensor MR imaging (DTI) analysis enables reconstruction of nerve fiber tracts through non-invasive quantification of the diffusion characteristics of water molecules along the three principle orthogonal diffusion directions [19]. DTI has been suggested to be sensitive enough to illustrate alterations of tissue microstructure [20], such as WM changes. The overall diffusivity and degree of directionality of diffusion in a tissue can be quantified using mean diffusivity (MD) and fractional anisotropy (FA), respectively [21]. In addition, directional diffusivities such as axial diffusivity (AD) and radial diffusivity (RD) are more specific to underlying biological processes, such as myelin and axonal changes [22]. A previous DTI study has shown that patients with PD have decreased FA in the WM tracts of the right inferior fronto-occipital fasciculus, left superior longitudinal fasciculus, and left body of the corpus callosum [23]. Han et al. [24] reported that patients with PD exhibit significantly greater FA values in the cingulate regions than do control subjects, and FA was found to be positively correlated with clinical severity. Recently, subtle changes of fronto-temporal WM integrity were found after remission, which might represent the neural correlates of treatment effects in PD [25]. Even though a few DTI studies have shown scattered findings of altered WM integrity, little is known about the WM architecture in the frontal lobe of PD.

Previous studies have suggested that WM integrity could be related to PD pathophysiology and the frontal cortex might play an important role in the fear network. Given the above considerations, we hypothesized that patients with PD would show altered integrity of WM tracts in the frontal lobe compared to healthy controls, which has been shown in studies suggesting the association between WM integrity and anxiety. Subsequently, we further investigated the relationship between WM abnormalities and clinical severity of PD. In addition to FA, other diffusion measures such as AD, RD, and MD were included in our analysis to investigate WM integrity more specifically. GM and WM volume analysis was conducted to rule out possible volume changes in corresponding brain regions. According to our previous finding that comorbid depression may affect the WM integrity in PD [26], only PD without any other psychiatric comorbidity was investigated in this study. In order to reduce the effect on medication on the results, we included patients who had been on medication for just a few days before imaging.

## Materials and Methods

### Ethics Statement

The CHA Bundang Medical Center Ethics Committee approved this study. All study procedures complied with the CHA Bundang Medical Center Institutional Review Board regulations, Declaration of Helsinki, and principles of Good Clinical Practice. All participants had the cognitive capacity to understand the research protocol and gave their oral and written consent after receiving a full description of the study.

### Subjects and Clinical Assessment

Thirty-six unrelated patients with PD and 27 age- and sex-matched healthy control (HC) subjects were included in the study. All subjects were between 18 and 60 years of age, were of Korean descent, and were right-handed.

Patients with PD were recruited by advertising at the outpatient clinics of authors (BK, TKC, and SHL), and HC subjects were recruited by public advertisement between January 2011 and December 2012. Subjects included in this study partially overlap those previously described elsewhere; these included 14 patients and 26 HC [26], [27]. PD was diagnosed using the Structured Clinical Interview for DSM-IV Axis I disorders [28] as diagnosed by experienced psychiatrists. Subjects were excluded if they had any current diagnosis or lifetime history of Axis I or Axis II psychiatric disorders other than PD, and/or if they had contraindications to magnetic resonance imaging (MRI) including metal implants and major medical or neurological disorders. Prior to the start of the study, patients with PD began treatment with a selective serotonin re-uptake inhibitor such as paroxetine, or escitalopram and benzodiazepines as anxiolytics, including alprazolam, clonazepam, or diazepam, within 1 week.

All subjects in both the groups were assessed for anxiety levels by using the Beck Anxiety Inventory (BAI) [29], [30]. In addition, panic symptom severity was assessed using the Panic Disorder Severity Scale (PDSS) [31], [32] in patients with PD.

### MRI Acquisition

All scans were acquired on a 3-Tesla GE Signa HDxt scanner (GE Healthcare, Milwaukee, WI, USA) at CHA Bundang Medical Center, CHA University. Parameters for three-dimensional T1-weighted fast spoiled gradient recalled echo (3D T1-FSPGR) images were as follows: repetition time (TR)  = 16 ms, echo time (TE)  = 4.3 ms, flip angle  = 10°, field of view (FOV)  = 25.6 cm, matrix  = 256×256, slice thickness  = 1.7 mm, and isotropic voxel size  = 1×1×1 mm^3^. Diffusion-weighted images were acquired using an echo planar imaging sequence with the following parameters: TR = 17000 ms, TE = 108 ms, FOV = 24 cm, matrix  = 144×144, slice thickness  = 1.7 mm, and voxel size  = 1.67×1.67×1.7 mm^3^. A double-echo option was used to reduce eddy-current-related distortions. To reduce the impact of spatial distortions, an 8-channel coil and array spatial sensitivity encoding technique (ASSET; GE Healthcare) with a sensitivity encoding (SENSE) speed-up factor of 2 was used. Seventy axial slices parallel to the anterior commissure–posterior commissure line covering the whole brain were acquired in 51 directions with b = 900 s/mm^2^. Eight baseline scans with b = 0 s/mm^2^ were also acquired. DT values were estimated from the diffusion-weighted images by using the least-squares method (approximate scan time  = 17 min). There was no hardware or software upgrades on the scanner between the recruitment drives [26], [27].

### Image Processing and Analysis

Voxel-wise statistical analysis of the FA data was performed using Tract-Based Spatial Statistics (TBSS) version 1.2 implemented in FMRIB Software Library (FSL version 4.1, Oxford, U.K., http://www.fmrib.ox.ac.jk/fsl) according to the standard procedure described in detail below [33]. First, DTI preprocessing, including skull stripping using the Brain Extraction Tool and Eddy Current Correction, were performed in FSL [34]. Next, diffusion tensor values (three eigenvectors and three eigenvalues) were then calculated for each voxel using a least-square fit to the diffusion signal. Finally, individual FA, axial diffusivity (AD), radial diffusivity (RD), and mean diffusivity (MD) metrics were derived from the three-dimensional maps of the three eigenvalues using FMRIB's Diffusion Toolbox. AD was defined as the largest eigenvalue (L1), RD was calculated as the average of the two small eigenvalues (L2 and L3), and MD was calculated as the average of the three eigenvalues (L1, L2, and L3) [35].

All subjects' FA data were then aligned into the standard space (Montreal Neurologic Institute 152 standard) by using FMRIB's Nonlinear Image Registration Tool. Transformed FA images were combined and applied to the original FA map, and this resulted in a standard-space FA map. These FA images were averaged to create a mean FA image, which was then thinned (skeletonized) to create a mean FA skeleton, taking only the centers of the WM tracts. The skeleton was thresholded by FA >0.2 (TBSS default) to include only major fiber bundles and to restrict the analysis to WM tracts that had been successfully aligned across subjects. The exact transformations derived for the FA maps were then applied to the other diffusivity volumes (AD, RD, and MD) for matched processing of image volumes per subject.

Statistical analysis was performed voxel-by-voxel to detect regions with significant FA differences between the PD and HC groups by using non-parametric permutation tests with a correction for multiple comparisons with the FSL Randomise program [36]. To achieve accurate inference, including full correction for multiple comparisons over space, we used permutation-based non-parametric inference within the framework of the general linear model tested with 5000 permutations, and the significance level was set at *p*<0.05. Multiple comparisons were corrected with Threshold-Free Cluster Enhancement (TFCE) [37], which allowed us to avoid making an arbitrary choice of the cluster-forming threshold, while preserving the sensitivity benefits of cluster-wise correction. Furthermore, analysis of covariance was conducted to confirm whether the same WM differences between two groups would be found when controlling for the effect of age and sex. Significant WM clusters were identified using an MRI atlas of human WM anatomy [38]. After statistical analysis, we selected only clusters of more than 50 contiguous voxels from the result images in order to further reduce the possibility of false-positive results.

Correlation analyses were conducted to investigate whether regional differences in WM integrity were potentially associated with the variance in clinical symptom ratings in each group. To assess correlations, the DTI data were analyzed using the TBSS General Linear Model (GLM) regression analysis with BAI (PD and HC group) and PDSS (PD group) as a factor.

For brain volume analysis, image processing for volume analysis of GM and WM was performed on the Statistical Parametric Mapping (SPM) 5 software (Wellcome Trust Centre for Neuroimaging, UCL, London, UK; http://www.fil.ion.ucl.ac.uk/spm) using the voxel-based morphometry (VBM) 5 toolbox (http://vbm.neuro.uni-jena.de/vbm) run on MATLAB 7.9 (MathWorks, Natick, MA, USA). The two-dimensional DICOM files of each brain were organized into volumetric three-dimensional files as NIFTI-1 (http://nifti.nimh.nih.gov) format using the MRIcron software package (http://www.sph.sc.edu/comd/rorden/mricron). In VBM preprocessing, the converted files of T1 images were segmented into GM, WM, and cerebrospinal fluid (CSF) compartments and normalized using the unified model [39]. Then, voxel values were modulated by the Jacobian determinants derived from the spatial normalization, which resulted in decreases in total counts for brain structures for which the volumes had decreased after spatial normalization; the decrease in total counts was proportional to the degree of volume discounted. The final voxel resolution after normalization was 1 mm^3^. Finally, the modulated GM and WM partitions were smoothed with a 12-mm full width at half-maximum Gaussian kernel and used for statistical analysis. In addition, global GM, WM, and CSF volumes as well as total intracranial volumes were computed using the native-space tissue maps of each subject.

For group comparison of GM and WM volumes between the PD and HC groups, independent t-tests and analysis of covariance (ANCOVA) with covariates of no interest, such as age, gender, and intracranial volume (ICV) were used. The statistical threshold was set at P<0.05, with family-wise error (FWE) correction for multiple comparisons at cluster level.

## Results

### Sociodemographic Data

Patients with PD and HC subjects had no significant differences in age (t = 0.28, *p* = 0.78), sex (*χ^2^* = 0.005, *p* = 0.94), and education years (t = −1.24, *p* = 0.22). Clinical characteristics and scores for rating scales for patients with PD are shown in Table 1.

**Table 1:** Sociodemographic and clinical characteristics of participating subjects.

	Panic disorder (PD) patients (n = 36)	Healthy control (HC) subjects (n = 27)	*t* or *χ^2^* a	p
Sex, Male/Female	15/21	11/16	0.005	0.94
Age (years, mean±SD)	38.83±8.68	38.15±10.86	0.28	0.78
Education (years, mean±SD)	13.92±2.74	14.83±3.09	−1.24	0.22
Duration of illness (months, mean±SD)	50.24±70.83	NA		
Agoraphobia, yes	25	NA		
PDSS score (mean±SD)	12.47±5.77	NA		
BAI score (mean±SD)	25.89±12.35	2.11±3.40	11.01	<0.001
Medication status b				
SSRI equivalent dosage (mean ±SD, mg) c	5.56±1.28			
Benzodiazepine equivalent dosage (mean ±SD, mg) d	1.12±0.43			

BAI, Beck Anxiety Inventory; NA, not available; PDSS, Panic Disorder Severity Scale; SD, standard deviation; SSRI, selective serotonin re-uptake inhibitor.

a *χ* ^2^ statistics were used to analyze frequencies and *t* tests were used to test mean differences.

b Duration of medication before scan of all patients was within a week.

c The approximate equivalent oral doses to 10 mg escitalopram are given.

d The approximate equivalent oral doses to 1 mg lorazepam are given.

### TBSS Data

**Figure 1:**
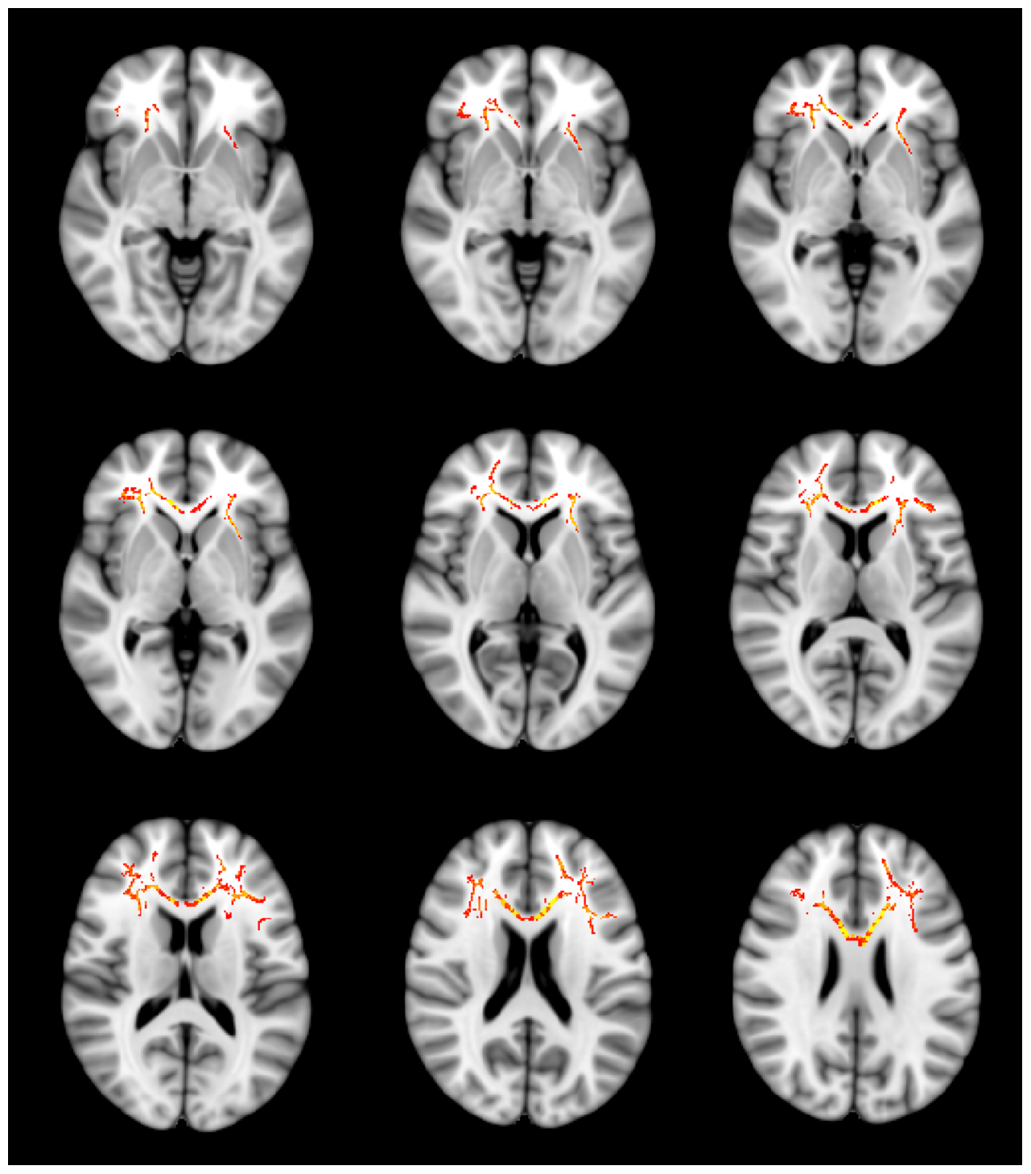
Regions of significant fractional anisotropy (FA) reduction in patients with panic disorder (PD) without comorbidity (N = 36) compared to healthy control (HC) subjects (N = 27). Voxels demonstrating significantly (threshold-free cluster enhancement, p<0.05 family-wise error corrected) decreased FA values for the PD group than for the HC group are shown in red-yellow (Z = −5 to Z = 25). Further cluster details are given in Table 2. Number of permutations was 5000. Left–right orientation is according to the radiological convention.

Compared to HC subjects, patients with PD exhibited significantly decreased FA values for frontal WM in both hemispheres. Results of TBSS analysis between the PD and HC groups showed three clusters of significant (*p*<0.05 TFCE-corrected) voxels on the WM skeleton. The largest cluster (3168 voxels) was located in the left hemisphere, containing the anterior corona radiata (frontal lobe WM) and body of the corpus callosum. The WM tracts of this cluster including the anterior corona radiata, extended from the internal capsule, radiate extensively in the frontal lobe, from the superior, middle, and inferior frontal gyri WM to the cingulum along with the corpus callosum. The second cluster (1860 voxels) was observed in the right hemisphere and comprised the anterior corona radiata radiating also in the frontal lobe, from the superior, middle, and inferior frontal gyri WM to the cingulum, approaching the genu of the corpus callosum. The last cluster (185 voxels) was adjacent to the second cluster in the right frontal lobe WM as well (Figure 1). For each cluster, the total number of voxels, peak coordinates, Z-value, anatomic location, and p value are listed in Table 2. Conducting group comparisons after controlling for the effects of age and sex did not alter the regions of significance. No regions of significantly increased FA values were found in patients with PD compared to HC subjects (comparison of FA values in whole-brain skeleton; *p* = 0.87 TFCE-corrected).

**Table 2:** Regions showing significant decreases of fractional anisotropy (FA) values in patients with panic disorder (PD) without comorbidity compared to healthy control (HC) subjects.

Cluster size (voxels)	Peak coordinates (mm)	Z	Anatomic locations	p a
3168	−25, 35, 1	4.30	Anterior corona radiata, left (frontal lobe white matter [WM])	0.028
	−16, 18, 27	4.16	Body of corpus callosum, left	0.019
	−22, 26, 0	3.95	Anterior corona radiata, left (adjacent to internal capsule)	0.027
	−17, −35, 34	3.80	Cingulum, left	0.033
1860	20, 38, 5	4.72	Anterior corona radiata, right (frontal lobe WM)	0.030
	9, 21, 18	4.19	Cingulum, right	0.035
	7, 23, 17	4.08	Genu of corpus callosum, right	0.035
185	21, −21, 46	3.95	Anterior corona radiata, right (frontal lobe WM)	0.045
	21, −20, 41	2.87	Cingulum, right	0.046

a Family-wise error corrected *p* value using the Threshold-Free Cluster Enhancement method.

No significant differences in MD, AD, and RD values were observed between the two groups. However, patients with PD showed increased RD values compared to HC subjects with a trend level of significance (*p* = 0.05 TFCE-corrected), located in clusters including the anterior corona radiata (frontal lobe WM), genu of the corpus callosum, and body of the corpus callosum in the left hemisphere, and the frontal WM in the right hemisphere.

**Figure 2:**
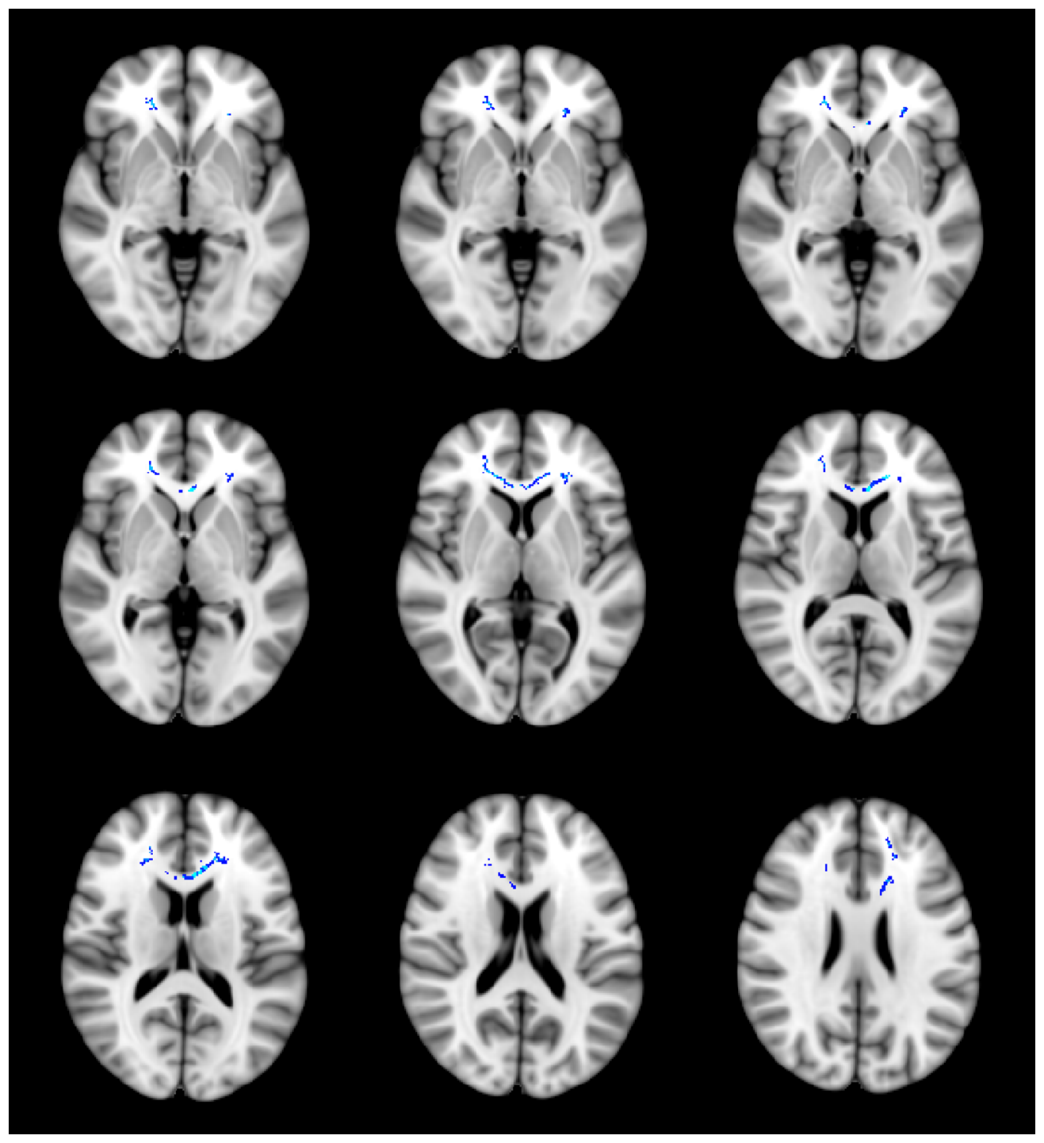
Regions of positive correlation between Beck Anxiety Inventory (BAI) scores and fractional anisotropy (FA) in patients with panic disorder (PD). Voxels demonstrating a significant positive correlation (threshold-free cluster enhancement, p<0.05 family-wise error corrected) between the BAI scores and FA values of the white matter clusters where a significant group difference was found are shown in blue-light blue (Z = −5 to Z = 25). Further cluster details are given in Table 3. Number of permutations was 5000. Left–right orientation is according to the radiological convention.

TBSS voxel-wise correlation analysis showed that the FA maps of patients with PD were positively correlated with BAI scores in several WM regions (*p*<0.05 TFCE-corrected), while there was no correlation between FA values and BAI scores in HC subjects (positive correlation, *p* = 0.21 TFCE-corrected; negative correlation, *p* = 0.97 TFCE-corrected). We compared these regions to the WM clusters showing significant group differences between patients with PD and HC subjects. The BAI score was shown to be positively correlated with the FA values in regions showing between-group differences, such as the frontal WM of both hemispheres, after multiple comparison correction (two measurements; Bonferroni correction, level of significance alpha  = 0.05/2 = 0.025) (Figure 2; Table 3). No significant correlation was found with the PDSS scores.

**Table 3:** Regions showing a significant positive correlation between the anxiety rating scale scores and fractional anisotropy (FA) in WM regions showing a significant between-group differences in patients with panic disorder (PD).

Anxiety rating scale	Cluster size (voxels)	Peak coordinates (mm)	Anatomical locations	p a
BAI	81	20, −8, 46	Anterior corona radiata, right (frontal lobe WM)	0.019
	62	−18, −11, 49	Anterior corona radiata, left (frontal lobe WM)	0.018
PDSS				NS

Note: There was no region of significant negative correlation between BAI scores and FA in patients with PD. Regions remained significantly after multiple comparison correction (2 measurements; Bonferroni correction, level of significance alpha  = 0.05/2 = 0.025) are shown.

BAI, Beck Anxiety Inventory; NS, non-significant.

a Family-wise error corrected *p* value using the Threshold-Free Cluster Enhancement method.

### VBM data

VBM analysis showed no significant difference in GM and WM volume between PD and HC subjects. Inclusion of age, gender, and ICV as covariates did not alter the results for group comparisons.

## Discussion

This study examined our hypothesis regarding the relationship of frontal WM integrity and PD. To our knowledge, these DTI findings represent the first report of extensive frontal WM alterations in patients with PD without comorbid conditions. The present results showed that PD might exhibit structural changes in WM integrity of the frontal lobe, which plays a major role in the fear network. In patients with PD, a relationship between frontal WM integrity and clinical severity of PD was found. Taken together, our findings suggest that PD is associated with microstructural abnormalities in specific WM circuits, most notably the WM of the frontal lobe. Further, we found an association between WM alterations and clinical severity. The results of this study could be related to aberrant frontal function and inadequate top-down control in PD. Since cognitive–behavior therapy (CBT) has been suggested to have the potential to facilitate frontal cortex control over the amygdala, these alterations in frontal WM integrity might be linked to the therapeutic effect of CBT in PD.

The present study indicates decreased WM integrity in patients with PD than in HC subjects; this finding is compatible with those of several studies. Abnormal integrity of various WM tracts connecting with the frontal cortex has been found in several anxiety disorders, such as generalized anxiety disorder [40], social anxiety disorder [41], and posttraumatic stress disorder [42]. Although our study replicated the changes of frontal WM integrity common across anxiety disorders, the findings of Lai et al. [23] were different from ours; those authors found reduced FA in a part of the right inferior fronto-occipital fasciculus and the left superior longitudinal fasciculus in patients with PD. While the subjects in Lai et al. 's study were first-episode, late-onset, and middle-aged (mean age, 47.03±10.63 years) individuals, our PD patients were mostly in their 30's with a history of panic symptoms. In addition, the age difference between the PD and HC groups of the two studies was different (this study, PD patients 38.83±8.68 years, HC 38.15±10.86 years, p = 0.78; the study by Lai et al., PD patients 47.03±10.63 years, HC 41.14±11.81 years, p = 0.08); this could have affected the results for comparison of WM integrity between the two groups. Thus, the inconsistency between the findings of the two studies might be attributable to the different characteristics of enrolled participants between these studies.

According to prior studies of traumatic brain injury, anxiety symptoms are associated with frontal WM lesions [43], [44]. In patients with social anxiety disorder, the integrity of frontal WM is also decreased [45]. Our finding of decreased FA in extensive frontal WM regions (WM tracts extended to the inferior, middle, and superior frontal gyri anteriorly, and mainly along the anterior corona radiata, approaching the cingulate gyrus and internal capsule in both the hemispheres) is in line with those of studies demonstrating compromised frontal function in PD [46], [47], as well as with imaging findings of reduced size [16], [18], metabolism [48], [49], and benzodiazepine receptor binding potential [50] of the frontal cortex. The anterior corona radiata, although not identified in the few previous DTI studies on PD, is fan-shaped WM in the frontal lobe connecting the limbic-thalamo-cortical circuitry [20], [51] and the prefrontal cortex gray matter areas associated with impaired top-down emotion regulation systems in PD. Previous studies have suggested that in PD, the frontal cortex cannot inhibit the hyperactivity of the anxiety-related neural circuitry [52], and several PD studies have revealed frontal cortical deactivation during anxiety provoked by pharmacological challenges [53], [54] or during spontaneous panic attacks [55]. Aberrant WM integrity of the frontal areas in this study may in fact reflect the frontal dysfunction seen in PD.

Altered WM integrity of the genu and body of the corpus callosum was also found to be associated with PD in the current study. Agenesis of the corpus callosum could induce various psychiatric symptoms, including anxiety [56], and size abnormality or atypical integrity of the corpus callosum has been suggested in anxiety disorders [23], [57], [58]. The altered integrity of the corpus callosum observed in this study might reflect aberrant interhemispheric communication in PD [59], which can cause dysregulation of balancing fear responses between both the hemispheres.

We found lower FA values along with a trend of higher, but not statistically significant, RD values for the extensive bilateral WM regions of the frontal lobe and corpus callosum among patients with PD. This finding is partly consistent with an altered neural network of fear conditioning [6] and abnormal activation [7] in the fear circuitry of anxiety disorders. The frontal cortex is believed to have a distinctive role in top-down control over anxiety [10], and the corpus callosum plays a role in interhemispheric communication. Altered WM integrity of the frontal lobe and corpus callosum observed in this study suggests dysfunction in controlling hyperactivity of the anxiety-related neural circuit along with interrupted communication between both the hemispheres in PD.

The physiological basis of pathologic alterations in FA remains unclear. In concordance with prior studies [23], [40], our study revealed lower FA values in several WM regions in patients with PD. In contrast, Han et al. 's study [24] reported increased FA values for the cingulate WM in PD. We think that the dissimilarities in subject characteristics and brain imaging analysis methods between two studies would affect the different results. While Han et al. did region-of-interest analysis on cingulate WM, we conducted whole brain voxel-wise analysis using TBSS. All PD subjects in Han et al. 's study were stabilized on medications, but our PD patients had been on medication for just a few days before imaging (within 1 week). Previously, our group demonstrated that the FA values were significantly higher across almost all the WM tracts in PD with comorbid depression compared to PD without comorbid depression [26]. The present results suggest that the FA values of patients with only PD without any comorbidity are lower than those of HC subjects. Although FA changes can be caused by various factors, lower FA of the frontal WM and corpus callosum in PD could reflect the decreased integrity of these areas.

A significant positive correlation has been observed between frontal WM integrity and clinical severity in PD [24], [27]. Further, correlational analyses have suggested that increased FA within the frontal WM in PD could be associated with higher anxiety. Furthermore, previous studies have shown a similar paradoxical positive correlation between clinical severity of conditions and neuroimaging measures, such as hallucination severity and FA in schizophrenia [60], harm avoidance level and brain dopaminergic function in Parkinson's disease [61], and anxiety severity and brain metabolism in OCD [62]. Our result for BAI scores may imply that the pattern of WM connectivity is determined, in part, by the secondary consequences of the primary pathological phenomenon in PD. Thus, the observed decrease in FA values for frontal lobe WM might reflect a compensatory mechanism, which, when effective, is associated with low anxiety in patients with PD. Having said that, we think that the observed positive correlation is difficult to explain based on current knowledge, and any conclusions made at this time will be premature.

To further investigate the structural differences between patients with PD and HC subjects, we examined other diffusion parameters such as AD, RD, and MD. AD decreases when axons are damaged, and RD increases in demyelination. MD provides an average measure of diffusion [35]. In addition to FA, these scalar measures are useful to investigate underlying biological processes of WM integrity, such as myelin and axonal changes. In general, FA decreases when RD increases and/or AD decreases. Even though we found a trend level of significance only for difference in RD between the groups, this alteration showed a large degree of overlap with the FA clusters. Decreased FA with increased RD was found in frontal WM and the corpus callosum of both hemispheres, suggesting increased water mobility perpendicular to the axons [63] in these regions. Possibly, this finding reflects a propensity for demyelination of the frontal lobe WM and corpus callosum in patients with PD.

Several limitations of this study should be noted. First, comparable to other studies, this study had a no more than moderate sample size in each group. Further studies should include a larger number of patients to obtain more accurate results. Second, the medication effect on brain WM integrity in the PD group could not be completely corrected. Although our patients had received medication only for a few days before the scan, undetected factors, other than the diagnosis of PD, might contribute to the observed WM alterations between the two groups. Third, patients were excluded from the current study if they presented with comorbidities other than PD. A fair number of patients with PD have comorbid conditions in clinical settings, which suggests that our patients were atypical to the general patients presenting clinically. However, since our goal was to determine the pathophysiological underpinnings of PD that are not attributed to the presence of comorbid conditions, we investigated the characteristics of WM integrity in “pure” PD. Fourth, the lack of a correlation between the PDSS scores and FA for frontal lobe WM might be a major limitation of this study. The PDSS score is a more specific measure of symptom severity in PD, whereas the BAI scores reflect broader anxiety. Thus, our finding of a significant positive correlation between FA values and BAI scores might indicate that the altered integrity of frontal WM is associated with anxiety in general rather than the panic-specific symptoms of PD. Lastly, a crossing fiber problem might affect the current results. The tensor model represents an independent, dominant direction, so the estimated orientation for voxels with complex fiber structure may be ambiguous or misleading. Future studies for addressing this crossing fiber problem are needed.

In conclusion, the present study implicates altered integrity of frontal WM in patients with PD without any comorbidity. Further, anxiety severity in patients with PD was positively correlated with their FA values of WM regions showing a significant intergroup difference. These findings provide evidence of altered WM integrity in the frontal lobe and suggest its contribution to PD anxiety symptoms. These changes could underlie the abnormal control of hyperactivity in the fear circuitry in patients with PD.
